# Analysis of the interactome of *Schistosoma mansoni* histone deacetylase 8

**DOI:** 10.1371/journal.pntd.0006089

**Published:** 2017-11-20

**Authors:** Stéphanie Caby, Lucile Pagliazzo, Julien Lancelot, Jean-Michel Saliou, Nicolas Bertheaume, Raymond J. Pierce, Emmanuel Roger

**Affiliations:** Univ. Lille, CNRS UMR 8204, INSERM U1019, CHU Lille, Institut Pasteur de Lille, Centre d'Infection et d'Immunité de Lille (CIIL), F-59000 Lille, France; University of Pennsylvania, UNITED STATES

## Abstract

**Background:**

Histone deacetylase 8 from *Schistosoma mansoni* (*Sm*HDAC8) is essential to parasite growth and development within the mammalian host and is under investigation as a target for the development of selective inhibitors as novel schistosomicidal drugs. Although some protein substrates and protein partners of human HDAC8 have been characterized, notably indicating a role in the function of the cohesin complex, nothing is known of the partners and biological function of *Sm*HDAC8.

**Methodology/Principal findings:**

We therefore employed two strategies to characterize the *Sm*HDAC8 interactome. We first used *Sm*HDAC8 as a bait protein in yeast two-hybrid (Y2H) screening of an *S*. *mansoni* cDNA library. This allowed the identification of 49 different sequences encoding proteins. We next performed co-immunoprecipitation (Co-IP) experiments on parasite extracts with an anti-*Sm*HDAC8 antibody. Mass spectrometry (MS) analysis allowed the identification of 160 different proteins.

**Conclusions/Significance:**

*Sm*HDAC8 partners are involved in about 40 different processes, included expected functions such as the cohesin complex, cytoskeleton organization, transcriptional and translational regulation, metabolism, DNA repair, the cell cycle, protein dephosphorylation, proteolysis, protein transport, but also some proteasome and ribosome components were detected. Our results show that *Sm*HDAC8 is a versatile deacetylase, potentially involved in both cytosolic and nuclear processes.

## Author summary

Using a target-based strategy to develop new drugs for the treatment of schistosomiasis we had earlier identified *Schistosoma mansoni* histone deacetylase 8 (*Sm*HDAC8) as essential for parasite development and survival in the mammalian host. Selective inhibitors of this enzyme show promise as lead compounds for drug development. However, the biological role of *Sm*HDAC8 has not been established. We identified the potential partner proteins and the processes in which it is involved by combining two methods: yeast two-hybrid screening and co-immunoprecipitation with mass spectrometry. These approaches yielded complementary sets of potential partner proteins that are actors in about 40 different cellular processes. These include known roles for the human counterpart of *Sm*HDAC8, like interactions with the cohesin complex and the cytoskeleton, as well as novel interactions such as with proteasomes and ribosomal proteins. Our results emphasize the implication of *Sm*HDAC8 in both nuclear and cytosolic processes and the versatility of this enzyme, suggesting why it is vital to the parasite and a promising drug target.

## Introduction

Schistosomiasis is a neglected tropical parasitic disease of major public health importance [1–3] caused by blood flukes of the *Schistosoma* spp., with 258 million people requiring treatment worldwide, and 780 million at risk of infection (http://www.who.int/mediacentre/factsheets/fs115/en/). There is currently no effective vaccine against schistosomiasis [4] and the treatment of the disease relies on a single drug, praziquantel (PZQ). Because of the intensive use of PZQ, field observations show the appearance of schistosome strains resistant to PZQ [5, 6]. Thus, the development of new drugs is imperative.

We have used a “piggy-back” strategy that consists in identifying orthologues of proteins already targeted in other pathologies, like cancer. Among these, we have chosen to target enzymes involved in epigenetic processes [7] and in particular, histone deacetylases (HDAC), which are among the most studied epigenetic targets. *Schistosoma mansoni* possesses three class I HDACs (*Sm*HDAC1, 3 and 8) and four class II HDACs, two class IIa and two class IIb enzymes [8, 9]. We have shown [10] that Trichostatin A (TSA), a pan-inhibitor of HDACs, induces hyperacetylation of histones, deregulates gene expression and induces the death of schistosome larvae and adult worms in culture. Schistosome HDACs are therefore promising targets for the development of new drugs against schistosomiasis, especially *Sm*HDAC8 [11, 12]. Indeed, it is the only schistosome class I HDAC for which the structure of its catalytic pocket differs significantly from that of the human orthologue [13], allowing the development of selective inhibitors that are toxic for schistosome larvae (apoptosis and death) and adult worms (changes in the reproductive organs, separation of worm pairs and arrest of egg laying) in culture [13–15]. Moreover, transcript knockdown of *Sm*HDAC8 leads to markedly reduced parasite viability and fecundity [13] suggesting that this enzyme is essential to parasite growth and development.

Human HDAC8 (hHDAC8) catalyzes the deacetylation of lysine residues within histone and non-histone proteins but its biological role has long remained elusive [11]. The two best-characterized non-histone substrates are the estrogen-related receptor (ERRα) and the structural maintenance chromosome 3 protein (SMC3). hHDAC8 interacts directly with ERRα *in vivo* and deacetylates ERRα *in vitro*, increasing its DNA binding affinity [16]. In the case of SMC3, a member of the cohesin complex, hHDAC8 is involved in its deacetylation that allows the recycling of the cohesin complex, and hHDAC8 mutations are linked with the Cornelia de Lange syndrome [17, 18]. Two recent studies using more systematic approaches identified novel hHDAC8 substrates. The first [19] detected seven proteins all of which are nuclear proteins, including SMC3, and this approach failed to identify histones or ERRα as substrates. The second study [20] detected 19 novel hHDAC8 substrates, not all of which were nuclear. Among them, the cohesin complex component SMC1A, but also all the protein substrates identified by Olson [19] were predicted by this approach. Clearly, each approach provides different information about the hHDAC8 substrates.

In order to characterize the protein partners of the 11 human HDACs, Joshi et *al*. [21] performed a global study of their interactions by establishing T-lymphoblast cell lines stably expressing Enhanced Green Fluorescent Protein (EGFP)-tagged hHDACs combined with proteomics and functional studies to identify hHDAC-containing protein complexes. They showed that in these cells hHDAC8 interacts with 15 proteins: four related to the cell cycle including SMC1A and 3, three related to protein and ion transport and 8 with other/unknown functions. However, although these proteins are members of hHDAC8-containing complexes, the methodology used cannot distinguish whether or not they interact directly with the enzyme. Moreover, human acetylome analysis [22] interestingly reveals that among these partner proteins, SMC1A, SMC3, SA2, SEC16A and NUP98 are acetylated but only SMC1A and SMC3 have been identified as hHDAC8 substrates [19, 20].

*Sm*HDAC8 shows major structural differences compared to hHDAC8, most obviously in the presence of insertions within the catalytic domain that form loops at the surface of the protein [13] and therefore represent potential surfaces for protein-protein interactions. We therefore sought to determine whether *Sm*HDAC8 interacts with the same or different proteins than hHDAC8. Since it is not possible to overexpress a tagged “bait” protein in schistosomes we decided to use two different methods to identify protein partners, yeast two-hybrid screening (Y2H) and co-immunoprecipitation experiments coupled to mass spectrometry (Co-IP/MS). The former technique characterizes direct protein-protein interactions and the latter identifies proteins that may interact directly or are members of protein complexes that interact with the target protein. They therefore yield different results, but in combination give an overall picture of the cellular processes in which *Sm*HDAC8 participates. Our results show that *Sm*HDAC8 is a versatile deacetylase, potentially involved in both cytosolic and nuclear processes, and contribute to the understanding of its status as a therapeutic target.

## Methods

### Yeast two-hybrid screening

A yeast two-hybrid (Y2H) *S*. *mansoni* adult worm (6 week-old male and female worms) cDNA library that consists of the Gal4-activation domain (Gal4-AD), amino acids 768–881, fused with *S*. *mansoni* adult worm cDNA was used. The cDNA library was constructed according to the manufacturer's instructions (Matchmaker Library Construction and Screening Kit, Clontech) using the pGADT7 plasmid containing the *LEU2* reporter gene. The cDNA library was transformed into *Saccharomyces cerevisiae* AH109 strain containing *HIS3*/*ADE2*/*LacZ* reporter genes, under the conditions recommended by the supplier (Yeast Protocols Handbook, Clontech). The cDNA library was screened with a bait construct corresponding to the Gal4-DNA binding domain (Gal4-DBD) fused with the full-length coding sequence of *Sm*HDAC8 (EF077628) [8], amplified using oligonucleotides *Sm*HDAC8 Fw (5'-GCTCGAATTCATGTCTGTTGGGATCG-3') and *Sm*HDAC8 Rw (5'- ACCTCGAGGATCCCATACCAGTTAAATTATA-3'), and cloned into the *Eco*RI and *Bam*HI restriction sites of the pGBKT7 vector bearing the *TRP1* reporter gene. The *S*. *cerevisiae* Y187 strain containing the *LacZ* reporter gene was transformed with the bait construct and mated with the AH109 strain overnight. After incubation, diploid yeasts were plated on selective medium lacking adenine, histidine, leucine and tryptophan and the plates were incubated at 30°C for 5 days. Positive clones were confirmed both by restreaking on selective medium and by a liquid LacZ assay (Yeast Protocols Handbook, Clontech).

### Extraction of plasmid from yeast cells and sequence analyses

Each selected positive clone was cultivated in medium lacking leucine. Cells were harvested by centrifugation (2,000 g, 20 min) and disrupted with glass beads. Plasmid extraction was performed with the Nucleospin plasmid kit (Macherey-Nagel) according to the manufacturer’s instructions. Plasmids extracted from yeast cells were then transformed in Subcloning Efficiency DH5α competent cells (Invitrogen). Prior to sequencing (Eurofins Genomics), about 400 individual clones were pre-screened by digestion with the restriction enzymes *Hind*III and *Eco*RV and electrophoresis on agarose gels in order to select clones with unique restriction profiles and weed out duplicates.

### Confirmation of selected clones

After extraction of plasmids, each clone was transformed in the AH109 strain to confirm that the interaction was robust. The *S*. *cerevisiae* Y187 strain was transformed with the bait construct *Sm*HDAC8 pGBKT7 and mated with the AH109 strain overnight. After incubation, diploid yeasts were plated first on selective medium lacking leucine and tryptophan and then on another higher stringency selective medium lacking adenine, histidine, leucine and tryptophan and the plates were incubated at 30°C.

### Bioinformatics analyses of proteins identified by Y2H

Sequence analyses (correction and alignment) were performed using Sequencher software (Gene Codes Corporation). Identification and functional annotation of *Sm*HDAC8 interactors was performed using Blast 2GO software [23].

### Ethics statement

All animal experimentation was conducted in accordance with the European Convention for the Protection of Vertebrate Animals used for Experimental and other Scientific Purposes (ETS No 123, revised Appendix A) and was approved by the committee for ethics in animal experimentation of the Nord-Pas de Calais region (Authorization No. AF/2009) and the Pasteur Institute of Lille (Agreement No. A59-35009).

### Parasite material

A Puerto Rican strain (NMRI) of *S*. *mansoni* is maintained in the laboratory using the intermediate snail host *Biomphalaria glabrata* and the definitive golden hamster host *Mesocricetus auratus*. *S*. *mansoni* adult worms were obtained by hepatic portal perfusion of hamsters infected six weeks previously [24].

### Antibody production

Purified recombinant *Sm*HDAC8 (a kind gift from M. Marek and C. Romier, IGBMC, Strasbourg, France [13]) was used to generate rat polyclonal antiserum. Male Lou Rats were injected i.p. with 50 μg of *Sm*HDAC8 with alum adjuvant in a total volume of 500 μL three times at two-week intervals. The rats were bled two weeks after the final injection. The monospecificity of the rat antiserum was controlled after *Sm*HDAC8 immunoprecipitation from *S*. *mansoni* protein extract (see section below) and western blotting (S1 Fig).

### Extraction and co-immunoprecipitation of proteins expressed in adult *S*. *mansoni*

Three independent experiments were performed as follows. Adult worms (50 couples) were suspended in 500 μL of lysis buffer (20 mM Tris HCl pH 7.4, 50 mM NaCl, 5 mM EDTA, 1% Triton and protease inhibitors), crushed with a Dounce homogenizer and sonicated ten times for 30 s (maximum power, Bioruptorplus, Diagenode). After centrifugation, at 10,000 *g* for 10 min at 4°C, immunoprecipitation of *Sm*HDAC8 was performed using the Pierce Crosslink Immunoprecipitation Kit (Thermo Scientific) according to the manufacturer’s instructions. Briefly, the protein lysate (500 μL) was pre-cleared by incubation with 20 μL of IgG from rat serum crosslinked to protein-L Agarose beads (Thermo Scientific) for 2 h at 4°C on a rotator. Then, pre-cleared lysate was collected after centrifugation, at 1,000 *g* for 1 min at 4°C, and incubated overnight at 4°C on a rotator, with 1 μL of anti-*Sm*HDAC8 antibodies or 1 μL of IgG from rat serum as a control, bound to protein-L Agarose beads.

### Mass-spectrometry proteomic analysis

Protein samples were denatured at 100°C in 5% SDS, 5% β-mercaptoethanol, 1 mM EDTA, 10% glycerol, and 10 mM Tris pH 8 buffer for 3 min, and subsequently fractionated on a 10% acrylamide SDS-PAGE gel. Electrophoretic migration was stopped when the protein sample had entered 1 cm into the separating gel. The gel was labelled briefly with Coomassie Blue, and five bands, containing the whole sample, were cut out. Digestion of proteins in the gel slices was performed as previously described [25].

Separation of the protein digests was carried out using an UltiMate 3000 RSLCnano System (Thermo Fisher Scientific). Peptides were automatically fractionated onto a commercial C18 reversed phase column (75 μm × 150 mm, 2 μm particle, PepMap100 RSLC column, Thermo Fisher Scientific, temperature 35°C). Trapping was performed during 4 min at 5 μL/min, with solvent A (98% H_2_O, 2% ACN (acetonitrile) and 0.1% FA (formic acid)). Elution was carried out using two solvents A (0.1% FA in water) and B (0.1% FA in ACN) at a flow rate of 300 nL/min. Gradient separation was 3 min at 5% B, 37 min from 5% B to 30% B, 5 min to 80% B, and maintained for 5 min. The column was equilibrated for 10 min with 5% buffer B prior to the next sample analysis.

Peptides eluted from the C18 column were analyzed by Q-Exactive instruments (Thermo Fisher Scientific) using an electrospray voltage of 1.9 kV, and a capillary temperature of 275 °C. Full MS scans were acquired in the Orbitrap mass analyzer over the m/z 300–1200 range with a resolution of 35,000 (m/z 200) and a target value of 5.00E + 05. The ten most intense peaks with charge state between 2 and 4 were fragmented in the HCD collision cell with normalized collision energy of 27%, and tandem mass spectra were acquired in the Orbitrap mass analyzer with resolution 17,500 at m/z 200 and a target value of 1.00E+05. The ion selection threshold was 5.0E+04 counts, and the maximum allowed ion accumulation times were 250 ms for full MS scans and 100 ms for tandem mass spectrum. Dynamic exclusion was set to 30 s.

### CoIP/MS proteomic data analysis

Raw data collected during nanoLC-MS/MS analyses were processed and converted into *.mgf peak list format with Proteome Discoverer 1.4 (Thermo Fisher Scientific). MS/MS data were interpreted using search engine Mascot (version 2.4.0, Matrix Science, London, UK) installed on a local server. Searches were performed with a tolerance on mass measurement of 0.2 Da for precursor and 0.2 Da for fragment ions, against a composite target decoy database (25,970 total entries) built with the *S*. *mansoni* Uniprot database (taxonomy id 6183, 12,861 entries) fused with the sequences of recombinant trypsin and a list of classical contaminants (124 entries). Up to one trypsin missed cleavage was allowed. For each sample, peptides were filtered out according to the cut-off set for protein hits with one or more peptides longer than nine residues, an ion score >30, an identity score >6, leading to a protein false positive rate of 0.8%.

### Accession numbers

Human HDAC8 (UniProtKB Q9BY41)
Human ERRα (UniProtKB P11474)
Human SMC3 (UniProtKB Q9UQE7)
Human EF1α1 (UniProtKB P68104)
Human SMC1A (UniProtKB Q14683)
Human SA2/STAG2 (UniProtKB Q8N3U4)
Human SEC16A (UniProtKB O15027)
Human NUP98 (UniProtKB P52948)
Human RAD21 (UniProtKB O60216)
Human PA2G4/Ebp1 (UniProtKB Q9UQ80)
Human CtBP (UniProtKB Q13363)
Human MBD2 (UniProtKB Q9UBB5)
Human RbAp46 (UniProtKB Q16576)
Human RbAp48 (UniProtKB Q09028)
Human MTA2 (UniProtKB O94776)
Human Cortactin (UniProtKB Q14247)
Human Anillin (UniProtKB Q9NQW6)
Human Gelsolin (UniProtKB P06396)
Human CapZ (UniProtKB P52907)
Human profilin (UniProtKB P07737)
Human Arp2/3 complex 1B (UniProtKB O15143)
Human Arp2/3 complex 2 (UniProtKB O15144)
Human Arp2/3 complex 3 (UniProtKB O15145)
Human Arp2/3 complex 4 (UniProtKB P59998)
Human RhoA (UniProtKB P61586)

## Results and discussion

The aim of our study was to identify *Sm*HDAC8 partners in order to better apprehend its biological role and function. We used here two strategies to characterize the *Sm*HDAC8 interactome.

The yeast two-hybrid (Y2H) screening using *Sm*HDAC8 revealed a large number of positive clones, of which we sequenced 137 after the prescreening step. After manual correction and assembly using Sequencher, we characterized 49 different sequences, which were then identified and functionally annotated using Blast 2GO software (S1 Table).

Three independent co-immunoprecipitation (Co-IP) experiments were performed, using an anti-*Sm*HDAC8 antibody (named IP1, IP2, and IP3). As a control, we performed Co-IP with a rat IgG antibody alone in each experiment. Mass spectrometry (MS) of the Co-IP proteins identified 1,500 different proteins (S2 Table). A significant degree of variation between the proteins identified in each experiment was noted, for which several reasons can be invoked. The three parasite protein extracts were each obtained from a pool of *S*. *mansoni* adult worms of both sexes and not from homogeneous cell cultures. Variations in protein expression between worm batches could induce differences between the three Co-IP/MS experiments. Moreover, *S*. *mansoni* is a complex multicellular parasite and protein quantities can vary between the different cellular types within a given individual as well as between different worms. Therefore, to take account of this relative variability between each extract, we chose to pool the results obtain for the three Co-IP/MS experiments IP1, IP2 and IP3. The possibility that the observed variability may have been due to non-specific interactions of our anti-*Sm*HDAC8 antibody with other proteins can be discounted. A single band corresponding to the molecular weight of *Sm*HDAC8 was detected on western blots of the immunoprecipitated material (S1 Fig) and the only HDAC detected by MS in the immunoprecipitates was *Sm*HDAC8 (S2 Table). Of the 1,500 proteins for which peptides were detected we selected only those that fulfilled three criteria: (i) at least three peptides in the Co-IP experiment, (ii) with no more than two peptides in the control and (iii) with a spectral count ratio between Co-IP *Sm*HDAC8 and control of greater than 3. After, this selection step we obtained 160 different proteins that were considered good candidates as *Sm*HDAC8 partners (S2 Table).

**Fig 1:**
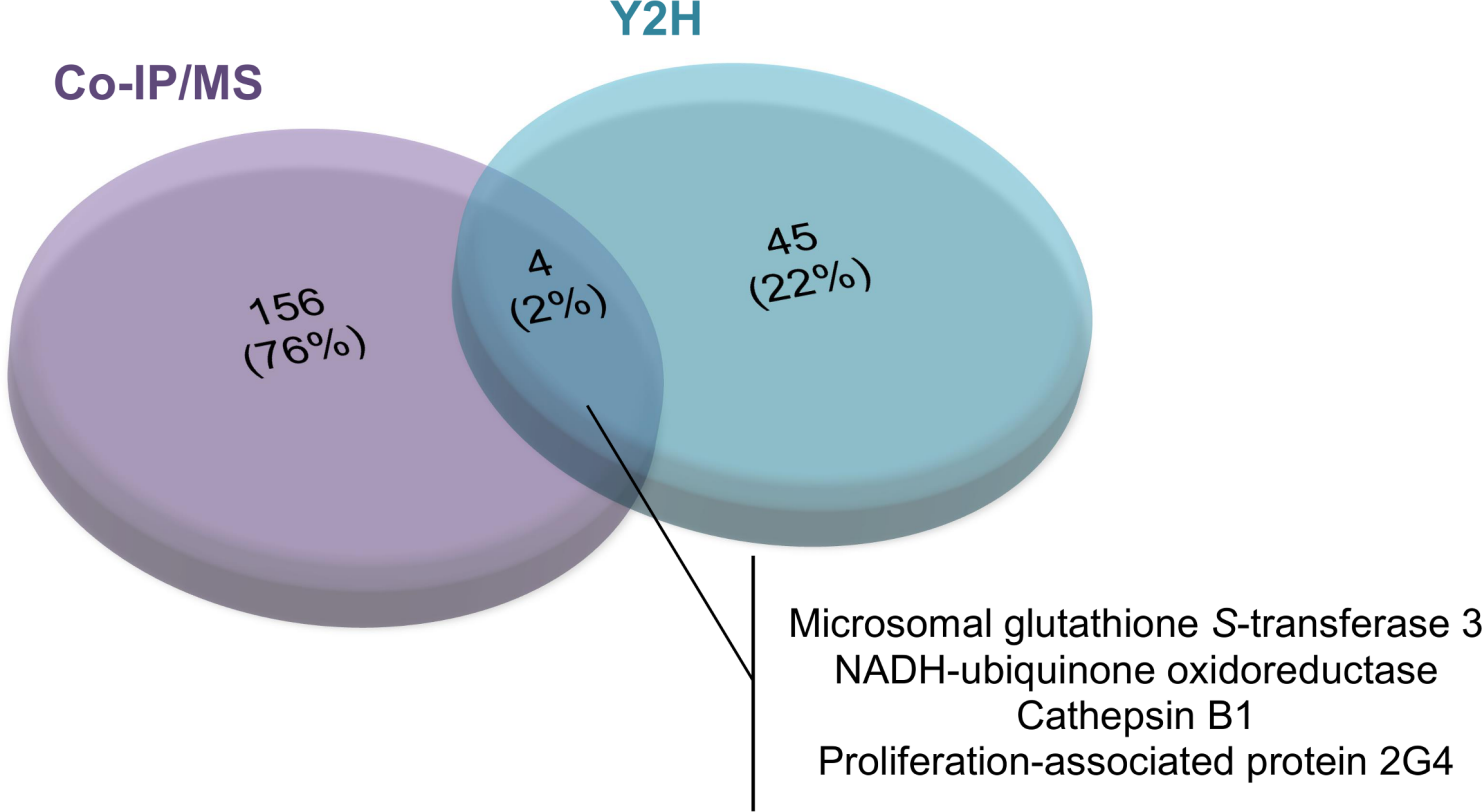
Venn diagram presenting the proteins identified by Y2H and Co-IP/MS as *Sm*HDAC8 interactors. Forty-nine and 160 different proteins were identified by Y2H and Co-IP/MS respectively. Four proteins are common between Y2H and Co-IP/MS: the Proliferation-associated protein 2G4, 38kDa (PA2G4, n°G4LXR6), Cathepsin-B1 (SmCB1, n°Q8MNY2), putative NADH-ubiquinone oxidoreductase (n°G4VK53) and microsomal glutathione *S*-transferase 3 (GST-3, n°G4VH65).

Among the proteins identified by these two approaches, four are common between Y2H and Co-IP/MS: the Proliferation-associated protein 2G4, 38kDa (PA2G4, n°G4LXR6), Cathepsin-B1 (SmCB1, n°Q8MNY2), putative NADH-ubiquinone oxidoreductase (n°G4VK53) and microsomal glutathione *S*-transferase 3 (GST-3, n°G4VH65) (Fig 1).

Among these proteins, two illustrate the previously characterized roles in HDAC-dependent processes and/or the potential involvement of acetylation. (i) PA2G4 is highly conserved in eukaryotes. The human member of this family, ErbB3 binding protein 1 (Ebp1) was identified as a putative downstream member of an ErbB3-regulated signal transduction pathway [26]. More particularly, the C-terminal region (300–372) of Ebp1, which is important for transcriptional repression, was shown to bind HDAC2 and inhibitors of HDACs significantly reduced Ebp1-mediated transcriptional repression [27, 28]. Like its human counterpart, the interaction between PA2G4 and *Sm*HDAC8 is mediated by its C-terminal moiety, corresponding to the fragment cloned in the Y2H screen. (ii) *Sm*CB1 is an essential gut-associated peptidase that digests host blood proteins as a source of nutriments (note that we also detected, but only with the Co-IP/MS experiments, cathepsin D and L2 which are also part of the gut peptidase network) and is also a drug target because enzyme inhibition induced severity phenotypes in the parasite [29]. Moreover, *Sm*CB1 is a promising vaccine candidate because its administration elicits protection against *S*. *mansoni* challenge infection in mice and hamsters [30, 31]. A direct link with HDACs has never been reported. Nevertheless, acetylome analysis from *Schistosoma japonicum* reveals that Cathepsin-B (n°Q7Z1I6) is acetylated on K241 and sequence alignments between *Sj*CB1 and *Sm*CB1 show that it is conserved.

The lack of convergence between Y2H screening and Co-IP/MS is perhaps not entirely surprising since they represent very different methods for interactome studies. Both strategies have weaknesses. In Y2H screening, direct interactions between two proteins are detected, but in some cases, these may be non-specific due to the juxtaposition of proteins or fragments that are never in contact within the cell. In the case of Co-IP/MS, some proteins cannot be identified because they are present in low quantities in the parasite extract and some proteins identified may be members of immunoprecipitated complexes and are not direct partners. The results obtained can therefore be considered as complementary and, taken together, provide an overall picture of the cellular processes in which *Sm*HDAC8 is involved.

**Fig 2:**
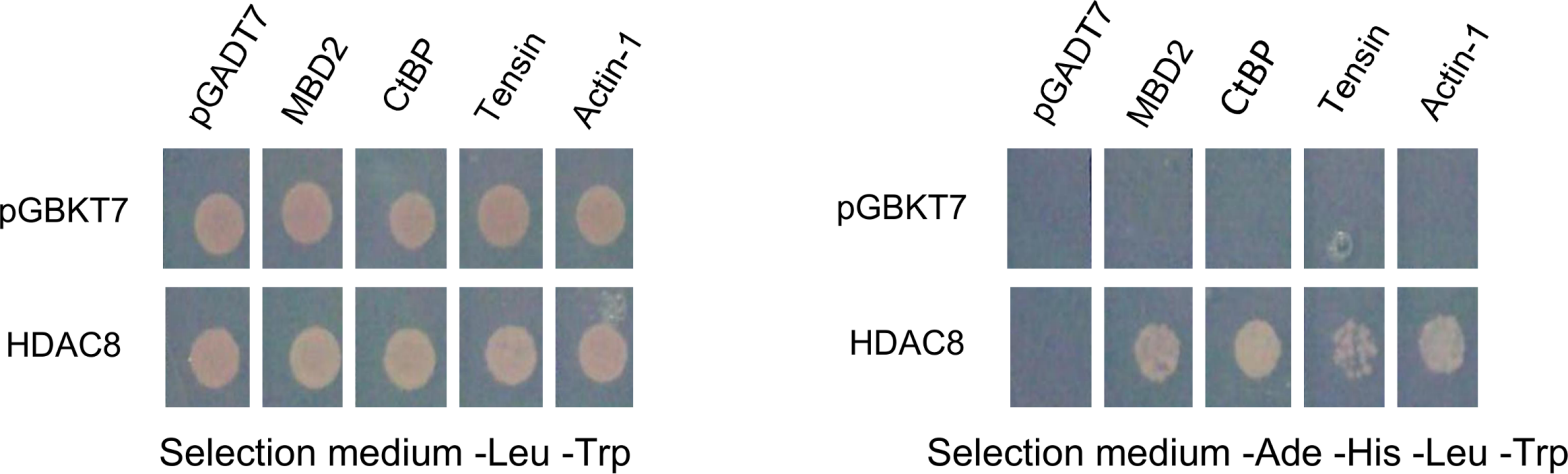
Confirmation of interactions by candidate-specific Y2H experiments. In order to confirm the screening results, the isolated clones CtBP, MBD2, tensin and actin-1 (cloned into pGADT7 vector) transformed in the AH109 strain were mated with the bait construct *Sm*HDAC8 pGBKT7 transformed in the Y187 strain. After incubation, diploid yeasts were plated on selective medium lacking leucine and tryptophan (left panel) and then on another high stringency selective medium lacking adenine, histidine, leucine and tryptophan (right panel) showing, as expected, that *Sm*HDAC8 interacts with the clones tested.

For some of the partner proteins identified only by Y2H and not by Co-IP/MS screening we carried out independent experiments to verify the interaction with *Sm*HDAC8. For instance, the binding of *Sm*CtBP, *Sm*MBD2, tensin and actin-1 proteins to *Sm*HDAC8 was verified by candidate-specific Y2H experiments. As expected, *Sm*HDAC8 indeed interacted with *Sm*CtBP and *Sm*MBD2, as well as the other two proteins (Fig 2). These results are in agreement with available data for the human orthologues. (i) The human C terminal binding protein (CtBP) family members appear to mediate transcriptional repression in a histone deacetylase (HDAC)-dependent manner [32]. Some human class I HDACs, HDAC 1, 2 and 3 [33–35] and class II HDACs (HDAC4 and 5) [36] are present in the CtBP1 nuclear protein complex, but the possible involvement of hHDAC8 was not investigated. (ii) The methyl-CpG binding domain protein 2 (MBD2) binds to methylated DNA and represses transcription through the recruitment of NuRD co-repressor complex [37]. The MBD2-NuRD complex contains a histone deacetylase core composed of HDAC1/2, RbAp46/48 and MTA2 [38–41]. The MBD2 protein possesses, among others, an intrinsically disordered region (IDR) able to recruit RbAp48, HDAC2 and MTA2 [42]. More particularly, it is possible that HDACs bind directly to the MBD2 IDR, because the human HDAC interactome study reveals a specific interaction between MBD2 and HDAC1/2 [21]. The Database of Protein Disorders (DisProt, www.disprot.org) [43] predicts an IDR domain located between the MBD and the C-terminal coiled-coil domains of *Sm*MBD2, which may be involved in *Sm*HDAC8 binding.

**Fig 3:**
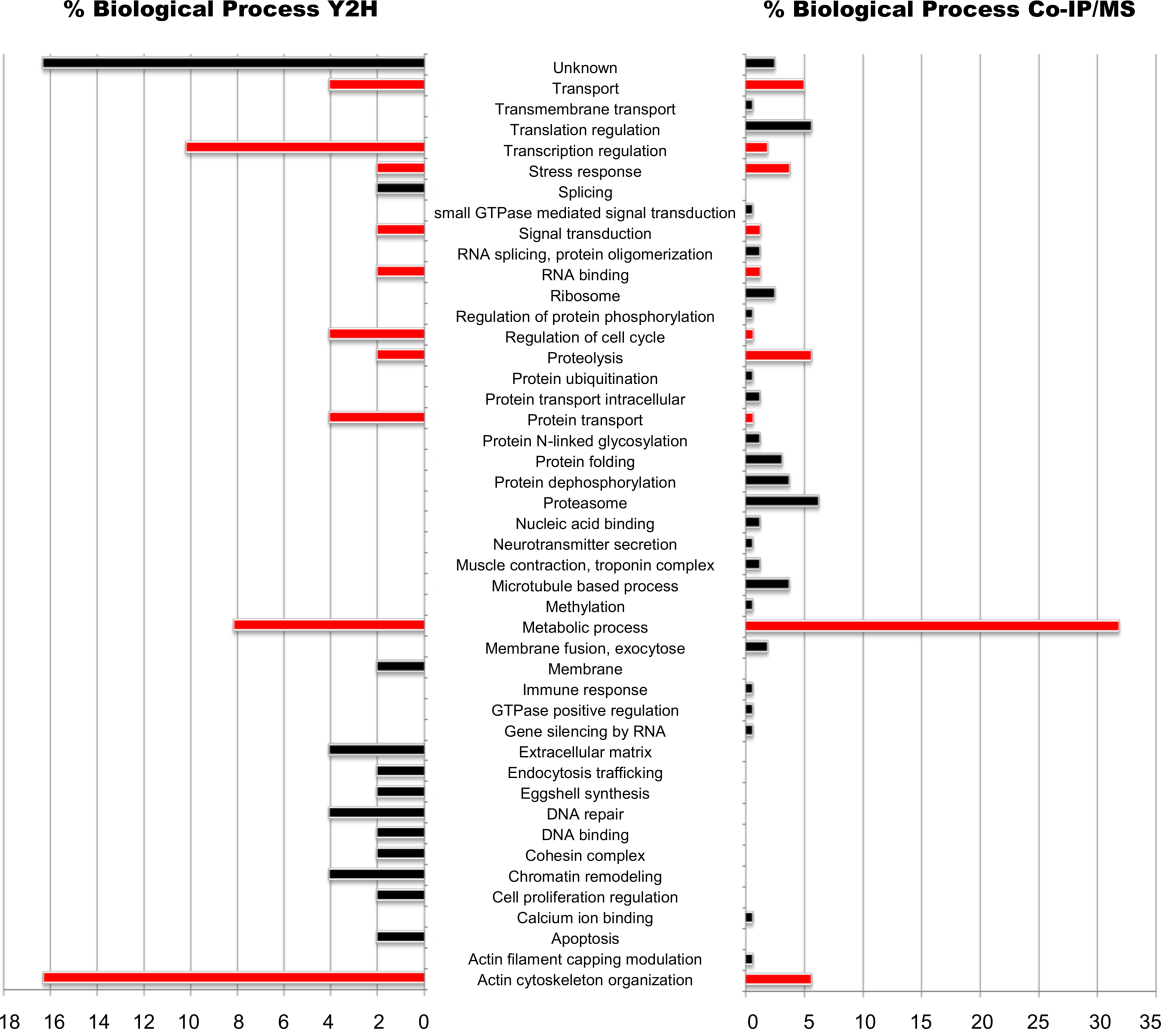
Biological processes involving the proteins identified by Y2H and Co-IP/MS as *Sm*HDAC8 interactors. Histogram (left for Y2H and right for Co-IP/MS) showing the biological processes in which the identified protein partners of *Sm*HDAC8 are involved. The processes were defined using the Blast2GO software. Bars represent the protein percentage belonging to one biological process. Red and black bars highlight common and specific processes between Y2H and Co-IP/MS, respectively.

Focusing on the biological processes predicted with the blast2GO software for each protein identified, among the 49 partners identified with the Y2H screening (S1 Table), seven were of unknown function, although one encoded a peptide including an EGF-like domain and an IgG-like domain. One further sequence corresponded to a hitherto unannotated gene. The remaining proteins are involved in 21 different biological processes (S1 Table), and those represented by the largest numbers of different sequences are cytoskeleton organization, transcription regulation, metabolism, transport, cell cycle regulation, DNA repair and chromatin remodeling (Fig 3).

The 160 proteins identified by Co-IP/MS (S2 Table) are involved in 33 different biological processes, and those represented by the most different sequences are metabolic process, cytoskeleton organization, proteasome, proteolysis, translation regulation, transport, protein dephosphorylation and stress response (Fig 3).

These results show that some of these processes (22%) are common between our different experiments, and in particular metabolism and cytoskeleton organization (Fig 3). Moreover, they suggest that *Sm*HDAC8 is a versatile deacetylase involved in both cytosolic (cytoskeleton organization, ribosome, proteolysis or proteasome) and nuclear (transcription regulation, DNA binding and repair, chromatin remodeling or cohesin complex) processes. This contrasts with the current knowledge of partners and substrates proteins of human HDAC8 (hHDAC8) that emphasizes nuclear functions. The global interactome study [21] that identified 15 partner proteins and the recent systematic studies of hHDAC8 substrates [19, 20] mainly identified nuclear partner proteins involved in the cohesin complex, transcriptional regulation and chromatin remodeling. Some extranuclear proteins were identified as partners, such as the Ca^2+^-dependent phospholipid binding protein Copine III [21] or substrates, such as the elongation factor 1 EF1α1 [20] but these were in the minority.

Here, the involvement of *Sm*HDAC8 in extranuclear functions is illustrated by the direct interaction between *Sm*HDAC8 and proteins involved in cytoskeleton organization like tensin, actin-1, actin 5c and Rho1 GTPase (S1 and S2 Tables). As previously mentioned, the binding of tensin and actin-1 proteins to *Sm*HDAC8 was verified by candidate-specific Y2H experiments. As expected, *Sm*HDAC8 indeed interacted with *Sm*Actin-1 and *Sm*Tensin (Fig 2). Interestingly, Waltregny *et al*. have shown that hHDAC8 interacts with smooth muscle alpha actin (but not beta-actin) to regulate cell contractility [44, 45]. Proteomic analyses have shown that all three human actin isoforms (alpha, beta and gamma) are acetylated [22, 46]. Moreover, the actin-associated proteins cortactin [47] and anillin [20] are hHDAC8 substrates. Several regulatory proteins of actin polymerization in human are also acetylated (gelsolin, CapZ, profilin and the Arp2/3 complex) [22] and we found some orthologues in our *Sm*HDAC8 Co-IP/MS analyses (gelsolin, septin and a subunit of the Arp2/3 complex, S2 Table). Actin dynamics are also controlled by small GTPases of the Rho family, like RhoA, notably in the formation of focal adhesions and stress fibers [48–50]. RhoA was not identified by Joshi *et al*. [21] as interacting with hHDAC8 and does not seem to be acetylated in human [22], although it was found to be acetylated in *Schistosoma japonicum* [51]. Here, our Y2H screen shows the direct interaction between *Sm*HDAC8 and *Sm*Rho1, the orthologue of human RhoA. Hence, it is possible that binding of *Sm*HDAC8 to *Sm*Rho1 could participate in the control of the *Sm*Rho1 pathway.

Among all the hHDAC8 substrates and partners identified [19–21], only Sec proteins, involved in endoplasmic reticulum protein secretory pathways were also found to interact with *Sm*HDAC8, although these are different and phylogenetically distinct proteins: Sec16A in human and Sec1 and Sec61β in *S*. *mansoni* (S2 Table).

Strikingly, we found no orthologues of the major hHDAC8 substrates/partners already characterized among the protein partners of *Sm*HDAC8. This is exemplified by members of the cohesin complex, which is notably responsible for the correct separation of sister chromatids into two daughter cells during mitosis [52]. The tripartite cohesin ring is formed by two SMC (structural maintenance of chromosome) proteins SMC1A and SMC3, and the α-kleisin subfamily protein RAD21 [53–55]. Both SMC3 and SMC1A were found in the hHDAC8 interactome [21], as is SA2 (an accessory protein that binds to hRAD21). In their study, hRAD21 was also detected, but at a non-significant level. Unexpectedly, we did not identify any of the cohesin complex members present in *S*. *mansoni* using our Co-IP/MS strategy contrary to Joshi *et al*. One possible explanation is that they used cells overexpressing EGFP-tagged hHDACs that generated a higher quantity of hHDAC8 protein than is present under physiological conditions. Correct MS identification depends strongly on the quantity of immunoprecipitated protein, and unfortunately, we are unable to increase *Sm*HDAC8 expression in *S*. *mansoni*. However, and interestingly, our Y2H screen shows a direct interaction of *Sm*HDAC8 with *Sm*RAD21. In order to confirm the Y2H screening result we carried out a candidate-specific Y2H experiment with the *Sm*RAD21 clone and, as expected, *Sm*HDAC8 interacts with *Sm*RAD21, suggesting that *Sm*HDAC8 forms an integral part of the cohesin complex and may have a central role in cell division, transcriptional regulation and DNA repair.

In conclusion, the protein partners of *Sm*HDAC8 identified by our Y2H library screen and Co-IP/MS experiments are all orthologues of proteins not previously identified as substrates or partners of human HDAC8. While we did not necessarily expect to detect substrates using these methodologies, it is striking that none of the partner proteins detected by Joshi *et al*. were among the *Sm*HDAC8 partners. We propose that the principal reason for this is that the methodology used in the previous study, with tagged HDACs (as bait to pull down protein complexes) expressed in a T-lymphoblast cell line, may address a limited subset of potential partners. In contrast, our Co-IP/MS study was performed on adult worm protein extracts and *S*. *mansoni* is a complex parasite encompassing a variety of cell types.

These Co-IP/MS approaches do not necessarily identify the proteins to which the HDACs physically bind because some proteins identified can be part of immunoprecipitated complexes. This is illustrated by the cohesin complex members identified by Joshi *et al*. (SMC1A, SMC3, and SA2) and that do not include RAD21, which only appeared as a non-significant binder. This is the reason why we also performed an Y2H screen using *Sm*HDAC8 in order to identify proteins that bound directly to *Sm*HDAC8. Our Y2H shows that *Sm*HDAC8 physically binds to *Sm*RAD21 and we suggest that the other cohesin complex components are pulled down by this interaction. Against this, it can be argued that the Y2H screen can identify non-specific interactions due to the juxtaposition of proteins or fragments that are never in contact within the cell. Again, this is why we have followed up the results of the initial screen with more detailed investigations of the interactions of two of the most interesting candidates, *Sm*RAD21 and *Sm*Rho1, allowing us to confirm that both are bona fide partners of *Sm*HDAC8.

Despite the fact that we did not identify the same protein partners as Joshi *et al*., it would be unwise to assume that all those identified in the Y2H screen and in the Co-IP/MS experiment are specific for the schistosome enzyme. It is probable that a number of their human orthologues will interact with hHDAC8 when investigated individually. Nevertheless, given the structural differences between the schistosome and human enzymes, and particularly the unstructured loops at the surface of *Sm*HDAC8, encoded by insertions in the catalytic domain sequence, it can be assumed that specific partners of the latter are present. Some of the proteins we identified have no human orthologues and have unknown functions. Further work will determine which of the partner proteins are schistosome-specific, how they interact with *Sm*HDAC8 and the role of these interactions within the parasite.

## Supporting information

S1 Fig Rat antiserum anti-*Sm*HDAC8 evaluation. The monospecificity of the rat antiserum was controlled after *Sm*HDAC8 immunoprecipitation from *S*. *mansoni* protein extract followed by western blotting using the same antibody (IP *Sm*HDAC8) and under the same conditions as used for the Co-IP/MS experiments. Control lanes are the *S*. *mansoni* adult worm extract used for the immunoprecipitation (input) and an immunoprecipitation with rat IgG (mock). The faint band at ~37kDa in the IP *Sm*HDAC8 lane is also present in the control (mock) lane.
(TIFF)

S1 Table *Sm*HDAC8 partners from Y2H screening. Identification and functional annotation of *Sm*HDAC8 interactors was performed using Blast 2GO software.
(XLS)

S2 Table *Sm*HDAC8 partners from Co-IP/MS analysis. Sheet “IP1-IP2-IP3 full list” contains the 1,500 different proteins identified from the three independent Co-IP/MS experiments IP1, IP2, IP3 respectively. Sheets “IP1 screen”, “IP2 screen” and “IP3 screen” contain the 57, 65 and 51 different proteins obtained after the selection step (cf. manuscript for details) for IP1, IP2 and IP3 respectively. Sheet “160 selected proteins” contains the 160 different proteins from IP1, IP2 and IP3.
(XLSX)
