# Supplementary figures and images for: Analysis of the interactome of Schistosoma mansoni histone deacetylase 8
Source: PLoS Negl Trop Dis. 2017 Nov 20;11(11):e0006089. doi: 10.1371/journal.pntd.0006089 (PMC5722368; doi:10.1371/journal.pntd.0006089)

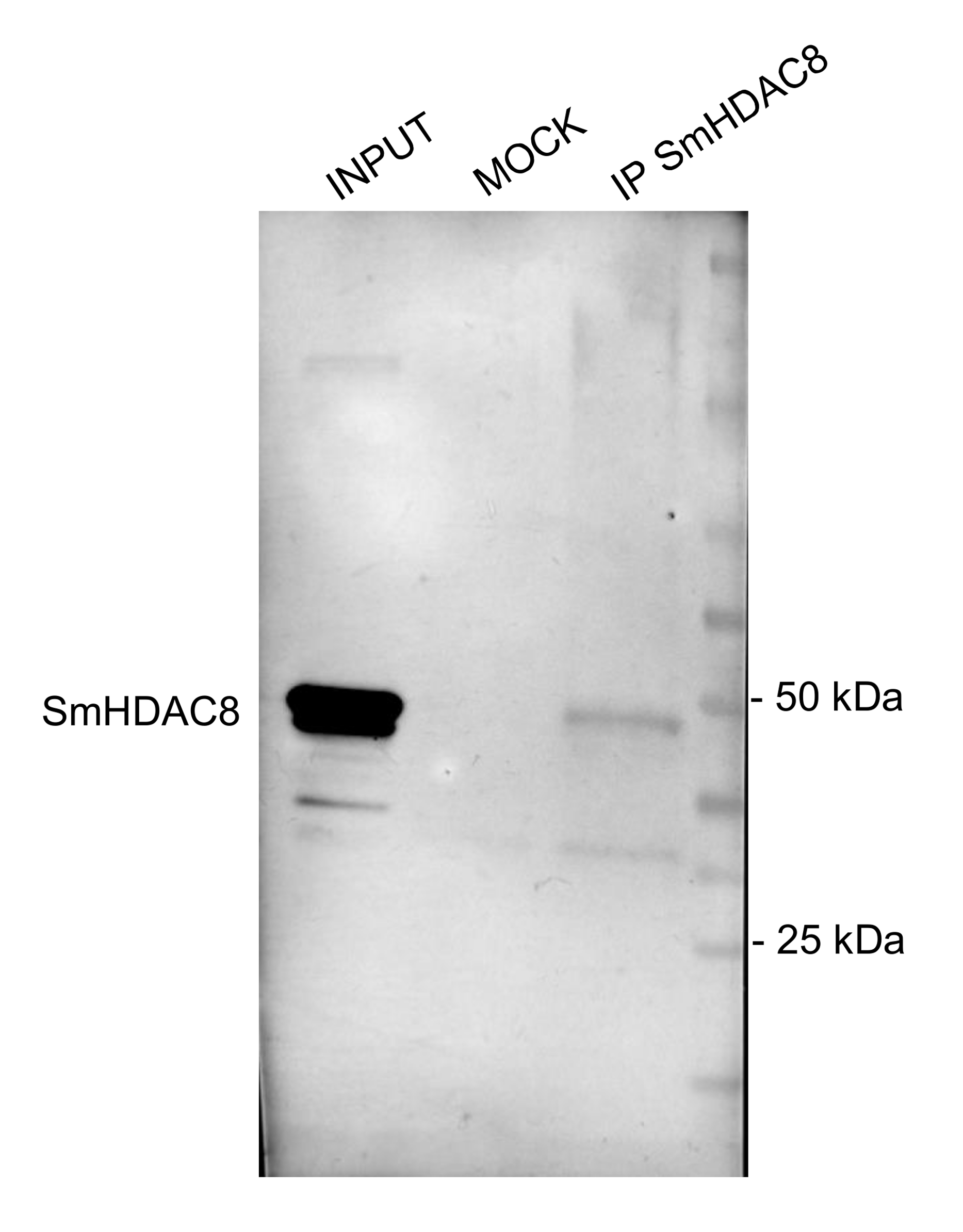

Supplement: S1 Fig — The monospecificity of the rat antiserum was controlled after SmHDAC8 immunoprecipitation from S. mansoni protein extract followed by western blotting using the same antibody (IP SmHDAC8) and under the same conditions as used for the Co-IP/MS experiments. Control lanes are the S. mansoni adult worm extract used for the immunoprecipitation (input) and an immunoprecipitation with rat IgG (mock). The faint band at ~37kDa in the IP SmHDAC8 lane is also present in the control (mock) lane. (TIFF) [file pntd.0006089.s001.tiff]
